# Immunotoxic Effects Induced by Microcystins and Cylindrospermopsin: A Review

**DOI:** 10.3390/toxins13100711

**Published:** 2021-10-08

**Authors:** Leticia Diez-Quijada, Maria del Monte Benítez-González, María Puerto, Angeles Jos, Ana M. Cameán

**Affiliations:** Área de Toxicología, Facultad de Farmacia, Universidad de Sevilla, Profesor García González n°2, 41012 Seville, Spain; ldiezquijada@us.es (L.D.-Q.); mariadelmontebg92@gmail.com (M.d.M.B.-G.); mariapuerto@us.es (M.P.); camean@us.es (A.M.C.)

**Keywords:** Microcystins, Cylindrospermopsin, immunotoxicity, in vitro, in vivo

## Abstract

Cyanotoxin occurrence is gaining importance due to anthropogenic activities, climate change and eutrophication. Among them, Microcystins (MCs) and Cylindrospermopsin (CYN) are the most frequently studied due to their ubiquity and toxicity. Although MCs are primary classified as hepatotoxins and CYN as a cytotoxin, they have been shown to induce deleterious effects in a wide range of organs. However, their effects on the immune system are as yet scarcely investigated. Thus, to know the impact of cyanotoxins on the immune system, due to its importance in organisms’ homeostasis, is considered of interest. A review of the scientific literature dealing with the immunotoxicity of MCs and CYN has been performed, and both in vitro and in vivo studies have been considered. Results have confirmed the scarcity of reports on the topic, particularly for CYN. Decreased cell viability, apoptosis or altered functions of immune cells, and changed levels and mRNA expression of cytokines are among the most common effects reported. Underlying mechanisms, however, are still not yet fully elucidated. Further research is needed in order to have a full picture of cyanotoxin immunotoxicity.

## 1. Introduction

Nowadays toxic cyanobacterial blooms are recognized worldwide as an emerging environmental threat because they can produce a sizeable number of secondary metabolites [1]. These secondary metabolites, called cyanotoxins, are produced as a consequence of an increase in cyanobacterial blooms resulting from anthropogenic activities, climate change and eutrophication [2,3]. Humans can be exposed to cyanotoxins in different ways, mainly orally, although inhalation and dermal exposure during recreational activities are also common [2]. Due to the toxic risks resulting from exposure to cyanotoxins, The World Health Organization (WHO) and the European Food Safety Authority (EFSA) have catalogued cyanobacteria as an emerging health issue [4,5].

Cyanotoxins can be classified according to their target organ. At present the main toxin groups are hepatotoxins, cytotoxins, neurotoxins, dermatoxins, and irritant toxins [6]. Among cyanotoxins, Microcystins (MCs) are cyclic heptapeptides composed of five common amino acids, plus a pair of variable L-amino acids [7]. These cyanotoxins act mainly by inhibiting the protein phosphatases 1 and 2A (PP1 and PP2A), and to date at least 279 MC congeners have been recognized [8]. Microcystin-LR (MC-LR) is the variant more frequently assessed, although some other variants (MC-RR, MC-YR) have been gaining more interest due to their toxicity [9,10,11,12]. Severe hepatotoxicity and numerous toxic effects including nephrotoxicity, neurotoxicity, reproductive toxicity and endocrine disruption have been reported for MCs [11,12,13,14,15,16,17,18]. Cylindrospermopsin (CYN) is a steady tricyclic alkaloid formed by a tricyclic guanidine moiety linked to hydroxy-methyl-uracil through a hydroxyl bridge [19]. CYN inhibits protein [20] and glutathione synthesis [21]. It is classified as a cytotoxin even though it can affect different organs [22]. It has also been shown to exhibit dermatoxic and neurotoxic activity, and CYN induces pro-genotoxicity via hepatic cytochrome P450-dependent mechanism [18,23,24,25,26,27]. Furthermore, these two cyanotoxins are also able to cause oxidative stress [28,29,30,31].

For both cyanotoxins, there is growing evidence that besides the above-mentioned toxicity, they can also have immunomodulatory potential acting in a dualistic way, inducing both immunostimulatory and immunosuppressive responses in the immune system [32]. Immunotoxicity could be defined as “any adverse effect on the components of and/or function of the immune system by a biological, chemical, or physical agent resulting from either direct or indirect actions and reflecting either permanent or reversible toxicity” [33]. The immune system is specialized in defense against pathogens, and it is composed of a collection of cells and tissues dispersed throughout the body [34]. This system is divided into two components closely connected with each other: innate and adaptive or acquired immunity. Innate immunity is possessed by all kinds of multi-cellular organisms; however, acquired immunity exists only in vertebrates [35]. The innate immune system is the first to respond to pathogens and does not retain memory of previous responses. It is mediated by cellular components such as granulocytes (neutrophils, eosinophils and basophils), macrophages, mast cells, dendritic cells and natural killer cells (NK). The cells involved in innate immunity recognize foreign substances such as bacteria with toll-like receptors (TLR), the receptors for innate immunity, and regulate the activation of other cells by the production of various cytokines, complement and acute phase proteins [35].

With respect to macrophages, they are important components of innate immunity against different pathogens and are involved in the pathology of numerous inflammatory diseases [36]. Macrophages stimulated by pro-inflammatory molecules release a variety of endogenous mediators such as nitric oxide (NO), reactive oxygen species (ROS), tumor necrosis factor alpha (TNF-α), and Interleukin-6 (IL-6), among others, that trigger an inflammatory reaction. Activation of the nuclear factor-κB (NF-κB), a dependent intracellular signaling pathway in macrophages, includes the activation of mitogen-activated protein kinases (MAPKs) [37]. When MAPK is the dominant signal activated in TNF, this signal is connected to inflammation, apoptosis or necrosis [38]. Other important cellular component of the innate immune system are neutrophils. They exhibit potent antimicrobial responses through various intracellular and extracellular mechanisms including the release of granules containing cytotoxic and antimicrobial enzymes, phagocytosis and the production of ROS and NO [39].

If a pathogen persists, despite the innate immune defenses, the adaptive immune system is recruited. The adaptive immune system is highly specific to a particular antigen, can provide long-lasting immunity and needs memory B and T lymphocytes (B and T cells), immunoglobulins (Igs), and major histocompatibility complex (MHC) [40,41]. The main role of B cells is to produce high affinity Igs against foreign antigens, and to act as a professional antigen presenting cell (pAPC) to present processed antigen to activate T cells. The T cell receptor (TCR) is always membrane bound and once stimulated via interaction with antigen presented by the pAPC, in the presence of co-stimulation, the T cell can be activated to function as a helper (CD4^+^) T cell, a regulatory (CD4^+^) T cell or a cytotoxic (CD8^+^) T cell [39,42].

Pioneer studies concerning the role of the immune system in the pathogenicity of cyanotoxins focused on MCs and reported that they could regulate the production of interleukin-1 (IL-1), TNF-α, and the induction of the synthesis of NO in macrophages [43,44,45]. It was observed that MCs had a clastogenic effect in human lymphocytes connected with chromosomal breakage in a dose-dependent manner [46]. Other approaches showed the apoptotic effect of cyanobacterial bloom extracts in rat hepatocytes and human lymphocytes [47], and demonstrated the potential genotoxic effect of these extracts on immunocytes, which was reflected by the DNA damage in human lymphocytes [48]. With respect to CYN, Terao et al. [49] described necrosis of lymphocytes of the thymus of mice giving a single dose of 0.2 mg/kg purified CYN intraperitoneally (i.p.). Similarly, degeneration and necrosis of cortical lymphocytes in the thymus and lympho-phagocytosis in rodents as experimental models were observed by Seawright et al. [50] and Shaw et al. [51]. Moreover, CYN can induce oxidative stress and a significant increase in the frequency of micronucleus (MN) in the presence of S9 fraction in human lymphocytes [26,52,53]. These processes can eventually lead to adverse responses in the immune system.

In recent decades, research on the potential impact of cyanotoxins on the immune system, especially in the case of MCs, has increased significantly. There are different studies in which cyanotoxins can impact both the function of blood cells of the immune system (such as macrophages, neutrophils, lymphocytes) [53,54,55,56,57,58,59,60,61], and the production of ROS and NO [35,37,55,62,63,64,65,66]. Moreover, several authors have focused on the study of transcription factors and cytokine production [37,61,62,66,67,68,69,70]. Recently, immunological gene and protein expression, immunohistochemical analyses and immunofluorescence staining by exposure to MC-LR have been studied both in vitro [60,71] and in vivo [59,72,73,74].

In this regard, taking into account the growing occurrence of cyanobacterial toxins and therefore its potential increased exposure, the fact that immunotoxicity is not considered a primary toxicity mechanism of MCs and CYN, and that immune system alterations could lead to important consequences in the organisms, it is of high interest to gain a full picture about the toxicity of these two types of cyanotoxins on the immune system. Thus, the aim of this review was to gather the available information to date in the scientific literature on the topic, including potential mechanisms involved and identification of data gaps. The information used was compiled after an extensive search of different sources in the public domain (PubMed, ScienceDirect, Scopus, Google Scholar) considering reports published in all time ranges.

## 2. Microcystins

Table 1, Table 2 and Table 3 show the different in vitro and in vivo studies that deal with the immuno-toxic effects induced by MCs.

### 2.1. In Vitro Studies

Most of the in vitro studies available in the scientific literature that focused on immuno-toxic effects of MCs (Table 1) have been performed with pure standards, especially in the case of MC-LR, the most frequently studied MC-congener. The exact mechanisms of immunotoxicity of MCs have not yet been fully elucidated. In this sense, the in vitro studies constitute an adequate and important tool that allows us to deepen our knowledge of MCs-immunotoxicity. Different cellular models have been employed so far to investigate MC-LR immunotoxicity, highlighting human lymphocytes i.e. [47,63,75,76] and neutrophils [53], as well as mammals’ macrophages. i.e. [43,62,68,77], because their results could be more representative of the effects and immuno-toxic mechanisms that may occur in humans. These reports evidenced the immunomodulatory potential of MC-LR. Thus, it decreased cell viability and induced cell death by apoptosis [47,76,78,79], altered the production of different cytokines or their mRNA expression [43,62,68,75,80,81,82,83], and induced a chemotactic effect [63,84].

Moreover, changes in inflammatory parameters have been reported not only in cells from the immune system but also in hepatic cells [81,82] and bovine Sertoli cells (from testis) [71,85].

Apart from MC-LR, other different MC-congeners have been investigated, such as MC-YR, [Asp3]-MC-LR, MC-RR, and MC-LA [55,63,77,79,80,86]. These reports did not show a clear differential response in comparison to MC-LR. Kujbida et al. [55] observed some different responses between MC-LR, -LA and -YR. Thus, human neutrophils’ viability was not altered by MC-YR treatment. Rat neutrophils also released significantly greater amounts of cytokine-induced neutrophil chemoattractant-2αβ (CINC-2αβ), only after incubation with MC-LR. However, and for example, extracellular ROS levels in human and rat neutrophils were similarly altered by the three MCs. These authors concluded that hepatic neutrophil accumulation is further increased by MC-induced neutrophil-derived chemokine. On the contrary, Yea et al. [80] observed that concanavalin A (ConA)-induced lymphoproliferative response was decreased by MC-YR, but no significant effect was observed in the MC-LR treatment. Considering the importance that minority MC congeners are acquiring due to their occurrence and toxicity [11,12], and the interest shown by international authorities such as EFSA [5], additional focused studies dealing with immune responses would be of interest.

An even more limited number of studies employed cyanobacterial bloom samples or crude extracts [47,76]. Of these, only the second compared the effects of crude microcystin-containing extracts, purified microcystin-containing and non microcystin-containing extracts and indicated that the purified extract (with MC-LR, MC-RR and MC-YR) induced the highest cytotoxicity and genotoxicity on human lymphocytes, and that other compounds had an influence on the results. Toxic effects of cyanotoxin mixtures are also worthy of research as they represent a more realistic exposure scenario, and can show antagonistic, additive or potentiation responses [87,88,89,90,91].

There is also a certain number of studies that use fish cells as experimental models [79,92,93,94,95,96,97]. These studies also report similar responses to those shown by mammalian models (decreased cell viability, apoptosis, changes in respiratory burst activity, altered cytokine mRNA expression, etc.). There is a single in vitro study by Zhang et al. [97] performed in carp (C. auratus) lymphocytes that evaluated the efficacy of the chemo-protectant quercetin in regulating the MC-LR induced apoptosis. The protective effects of natural antioxidants on fish immunotoxicity have also been evidenced in vivo [98].

**Figure 1:**
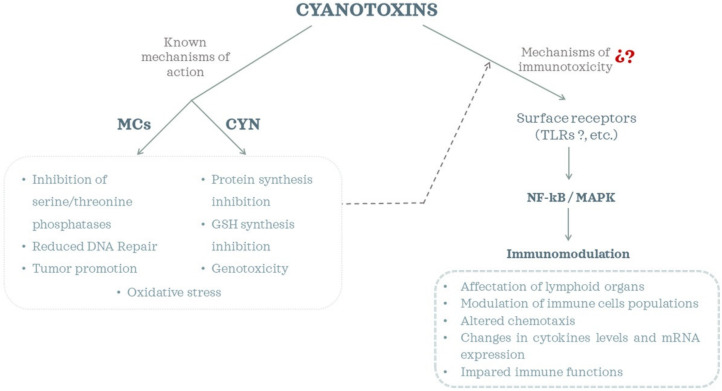
Cyanotoxins’ toxicity mechanisms and immunomodulatory effects.

Taken together, most of these studies explored changes in immune parameters, but the molecular pathways involved are not yet totally elucidated (Figure 1). There are some reports that suggested that MC-LR induced a dysfunction of the NF-κB and MAPK signaling pathways [82,83,85,97]. Inflammatory and immune responses are regulated by multiple signaling pathways, among which the NF-κB and mitogen-activated protein kinase (MAPK) signaling pathways, which include the extracellular signal-regulated kinase 1/2 (ERK1/2), p38, and c-Jun N-terminal kinase (JNK) pathways, are critical participants in cellular stress responses and modulate a variety of inflammatory responses [82,99,100]. Moreover, surface receptors (such as Toll-like receptors (TLRs)) have also been suggested to play a role in the observed immunomodulatory effects of MCs [71,83,85]. TLRs are known to transduce signals via adaptor proteins (e.g., MyD88), kinases (e.g., MAPKs, AKT) and transcription factors (e.g., NF-κB). However, recent studies by Hansen et al. [86] observed that MC-LR, -RR and -LA exhibited neither stimulatory nor inhibitory effects on cell lines that expressed specific TLRs, and they hypothesized that tested cyanobacterial toxins might initiate immune responses via NF-κB through interaction with other cell surface receptors. Therefore, more research efforts are still required to reveal the underlying mechanisms of cyanotoxin immunotoxicity.

### 2.2. In Vivo Studies

#### 2.2.1. Aquatic Organisms

The immuno-toxic potential of cyanotoxins in fish is an issue of interest taking into account the number of studies available in the scientific literature (Table 2). Rymuszka et al. [102] have already reviewed the topic and pointed out that the immuno-modulative effects of cyanotoxins on fish could result in higher susceptibility to diseases. From this report, additional studies have been performed, with special focus on MC-LR. There is a single study that compares the immunotoxicity potential of MC-LR versus an extract of a cyanobacterial bloom containing a similar level of MCs [101]; it concluded that the extract had greater suppressive effects on immune cells. It is known that environmental samples may include additional bioactive compounds (i.e., oligopeptides such as micro-ginins or aeruginosins, lipopolysaccharides, polar alkaloid metabolites and other unidentified metabolites [103,104] that have an influence on the toxicity observed [105].

From the results compiled, it is evidenced that MCs are able to induce pathological lesions in lymphoid organs and cells [72,74,106,107,108]. Interestingly, the toxic effect is recovered with time. Thus, Wei et al. [109] found that damaged lymphocytes were almost unobserved in the spleen and pronephros after 21 days of a single exposure to 50 µg/kg MC-LR body weight (b.w.) i.p. injection in grass carp, showing the spleen to have a higher sensitivity.

Cytokines gene expression by RT-PCR is the parameter most frequently investigated in aquatic animals (fish and shrimps) i.e., [72,101,110,111,112]. All of these studies evidenced that the exposure to MCs (MC-LR) affect the function of the immune system, resulting in immunomodulation. Moreover, this effect is independent of the method of exposure (i.p. injection, immersion, diet). Several authors agreed on a dualistic response of the fish innate immune system. Thus, fish immunity tends to proceed toward the direction of an immuno-stimulative response at low MC concentrations but toward the trend of immunosuppressive answer at high MC concentrations [59,69,106].

Another interesting finding is that there are substances such as L-carnitine that when used as functional feed additive could significantly inhibit the progression of MC-LR immunotoxicity [98]. The efficacy of this chemo-protectant on cyanotoxin-induced oxidative stress and histopathological changes was already known [113,114], but this is the first report that evidenced a positive influence also on the immune system. As oxidative stress triggers inflammatory responses, it can be hypothesized that other chemo-protectants that have shown to reduce MC-induced oxidative damage in fish such as N-acetylcysteine, Trolox, etc., [115,116] could also ameliorate immunotoxicity.

Different authors have also shown that MC-LR can induce cross-generational immunotoxicity not only in fish [70] but also in other aquatic organisms such as prawns [117]. Thus, Lin et al. [70] observed that after exposure up to 10 µg/L MC-LR for 60 days in zebrafish (F0 generation) and with/without continued exposure of the embryos until 5 days postfertilization, the F1 generation showed an upregulation on innate immune-related genes (tlr4α, myd88, tnfα, il1β) as well as increased proinflammatory cytokine content (TNF-α, IL-1β, IL-6). Moreover, the inflammatory response was higher in embryos with a continued exposure. Similarly, Sun et al. [117] reported that F1 offspring prawns (Macrobrachium nipponense) showed, among other effects, downregulation of immunity molecules (lysozyme, lectin3) and increased expression of innate immune-related factors (TLR3, MyD88), despite not being treated with MC-LR. These results indicate that parental exposure of aquatic organisms could jeopardize the immune homeostasis and healthy growth of larvae.

Overall, aquatic organisms are at high risk of cyanobacterial toxin exposure, and the immune system has been revealed as an important target, apart from the well-known hepatotoxicity of MCs.

#### 2.2.2. Mammals

Studies dealing with immunomodulatory effects of MCs on mammalian models are limited (Table 3). They have explored the effects of both bloom extracts containing MCs [54,67,119,120] and pure MCs, in this latter case restricted to MC-LR [60,61,73,121,122,123]. The preferred model is the mouse (70% of articles) followed by the rat (20%) and the rabbit (10%). Most used i.p. injection as the exposure route, and only three employed the oral route, two by drinking water [60,123] and a single one through the diet [120].

The research performed up to now has revealed that MCs impaired immune functions in different ways. Thus, a reduction of phagocytosis [54,122], changes in gene expression of different cytokines [67,73,123] and in their levels [61,73,119,122], modulation of cell populations [54,119,120], etc., have been reported (Figure 1). However, these effects appeared to depend on the doses or the method of exposure, etc., or to show variations in a transient manner. For example, Li et al. [121] observed severe damage in the spleen of Wistar rats exposed i.p., and a significant MC-LR accumulation in this organ. By contrast, Palikova et al. [120] reported no histological changes in the spleen or thymus of Wistar rats exposed through the diet. The compiled results also showed the importance of the immune system in the homeostasis of the whole organism, with effects not only in lymphoid organs but also in the reproductive system [60,73], bones [61], or jejunum [123].

Considering that the i.p. pathway is not the most relevant exposure route when considering potential effects in humans, a special focus on studies using the oral route is of interest. In this regard, Palikova et al. [120] performed a study in rats fed for 28 days with a diet containing fish meat with/without MCs and complex toxic biomass. They pointed out that toxic effects were less pronounced than expected (actually, no significant histological changes were observed in the spleen or thymus), and they suggested that other compounds of the diet could influence MC availability or have a positive effects on faster elimination. In any case, diets enriched with MCs (700 and 5000 mg total MCs per kg of feed, wet weight (w.w.)) influenced preferably innate parts of the immune system represented by NK cells and gamma-delta T cells, which play a role as a bridge between adaptive and innate immune response. On the other hand, rats fed with fish from a locality with heavy cyanobacterial bloom mainly showed changes in proportions of Th, Tc and double-positive T cells which represent cells of the adaptive immune system. The level of MCs in this type of diet was very low, so these effects were probably caused by the presence of other substances contained in the bloom.

Chen et al. [60] and Cao et al. [123] used mice exposed to MC-LR through drinking water but selected a different experimental design and focused on different target organs. Thus, they exposed mice to a concentration range of 1–30 μg/L MC-LR for 180 consecutive days and explored the outcome in the testes. They found that MC-LR treatment induced significant enrichment of macrophages in the testes and that these macrophages promoted Leydig cell apoptosis. Additionally, expression levels of macrophage activation marker protein TNF-α in the testes were significantly up-regulated. Similarly, mRNA levels of IL-6, monocyte chemoattractant protein-1 (MCP-1), Axl, and MerTK (tyrosine kinase receptors) were also increased at different concentrations. Cao et al. [123] focused their study on the jejunal microstructure and the expression level of inflammatory-related factors in this organ. They used mice exposed to 1–120 μg/L MC-LR for 6 months and observed an alteration in the expression levels of different inflammation-related factors depending on the concentration. Interestingly, these authors showed that even at 1 μg/L MC-LR, which is the current guidance value in water [124], MC-LR was able to alter immunologic parameters in mice.

Changes in hematological parameters, activation of neutrophils and macrophages, and alteration and activation of lymphocytes, were the specific effects on immune functions identified for MCs in mammals by Lone et al. [125]. As reports included in Table 3 evidence, little progress has been made so far in trying to decipher MCs’ immunotoxicity.

Thus, further research is required to understand the immunomodulatory effects of cyanotoxins. Particularly, mammalian studies with relevant exposure scenarios would be welcome to try to interpolate the potential consequences in humans.

## 3. Cylindrospermopsin

Among the effects induced by CYN, the toxin may also exhibit immunotoxicity [126], although studies focused on this pathogenicity are very scarce [25] (Table 4). The first work in which pure CYN was classified as a potential immuno-toxicant was reported in human peripheral blood lymphocytes. In this experimental model, CYN induced a significant inhibition of their proliferation [53]. Previously, Seawright et al. [50] had already provided hints of the immunomodulatory effects of CYN as they observed histopathological alterations (atrophy) in the lymphoid organs (thymus and spleen) in mice orally exposed to a *C. raciborskii* culture. This is actually the only report of CYN immunotoxicity in vivo.

Poniedzialek et al., have performed an extensive investigation of CYN effects on human immune cells, mainly lymphocytes [53,56,57,58,127]. These studies showed that CYN inhibited cell proliferation [53,56,58], reduced viability, altered cell cycle [56], and induced changes in oxidative stress parameters in lymphocytes [127]. Moreover, it decreased the oxidative burst capacity of neutrophils [57], cells that play an important role in non-specific immune response and organism resistance, specifically in anti-bacterial resistance. These authors also demonstrated that both CYN and non-CYN producing C. raciborskii extracts can exhibit immunomodulatory potencies. Thus, human lymphocytes in general were more resistant than neutrophils after exposure to cell-free extracts. Nevertheless, their short-term exposure resulted in a significantly increased rate of apoptosis and necrosis, whereas CYN did not induce similar effects. The effect of the non-CYN extracts on T- lymphocyte proliferation was not as pronounced as for CYN, suggesting that the cells can partially overcome the toxic effects induced by C. raciborskii exudates, or that the metabolites are degraded due to their lower stability than that of CYN.

Apart from human cells, pure CYN was reported to impair and decrease the function of phagocytic cells in the CLC cell line from fish (Cyprinus carpio) [128] and in phagocytic cells isolated from head kidneys of the same fish species. In this model system, cytotoxicity was observed, as well as a decreased phagocytic activity, changes in actin cytoskeletal structures, production of ROS and NOS, and altered expression of specific genes of proinflammatory cytokines [66].

Moosova et al. [37] investigated whether CYN, either alone or in combination with the immunomodulatory agent lipopolysaccharide (LPS), could affect murine macrophage-like RAW 264.7 cells, the model of mammalian innate immunity. They found that CYN induced the TNF-α production and pro-inflammatory phenotype in macrophages and also increased ROS that trigger an inflammatory response. Moreover, it can synergistically potentiate the stimulation of macrophages by LPS. It is known that LPS stimulates the phagocytic cells via Toll-like receptor 4 (TLR4), resulting in production of proinflammatory cytokines as a result of NF-κB and MAPK activation. It has also been suggested that cyanotoxins interact with TLRs, key triggers of inflammatory processes [37,77,83]. However, recently Hansen et al. [86] found that CYN (and other cyanotoxins) did not directly interact with human TLRs in either an agonistic or antagonistic manner. Thus, reports of cyanotoxin-induced NF-κB responses likely occur through different surface receptors.

As has been evidenced, the information available regarding the immunomodulatory effects of CYN is very limited, even more sp the elucidation of the molecular mechanisms involved (Figure 1). Further research is required on this issue to develop effective prevention and intervention strategies against CYN toxicosis.

## 4. Final Considerations

Cyanotoxins (MCs and CYN) have been shown to induce immunomodulatory effects, with impact on both innate and adaptive immunity. They induced pathological alterations in immune cells and organs, altered immune cells (macrophages, neutrophils, lymphocytes, etc.), changed levels and mRNA expression of cytokines, and altered chemotaxis, among other effects. However, the mechanisms underlying these responses are far from being well understood. This is due to the still limited number of studies that focus on this topic, particularly for CYN. Different studies have proposed a TLR dependent NF-κB activation by MCs. TLRs are known to transduce signals via adaptor proteins (e.g., MyD88), kinases (e.g., MAPKs, AKT) and transcription factors (e.g., NF-κB). However, recently Hansen et al. [86] reported that neither MCs nor CYN interacted with human TLRs in either an agonistic or antagonistic manner. They proposed that other cell surface receptors such as peptidoglycan recognition proteins, scavenger receptors, receptor tyrosine kinases or cytokine receptors could be involved. Moreover, there are other receptors involved in the dysfunction of the immune system. This is the case with peroxisome proliferator-activated receptors (PPARs) that belong to the nuclear steroid receptor superfamily [129], although the interaction of cyanotoxins with these has not been explored. Hansen et al. [86] also pointed out that organic anion-transporting polypeptides (OATPs) could potentially activate NF-κB in a non-specific manner. OATPs are a well-known family of membrane transporters used by MCs to enter the cell. In this regard, different studies have reported the presence of OATPs in lymphoid organs such as spleen and thymus [130], immune cells such as monocytes and macrophages [131], lymphocytes [132], etc. However, Adamovsky et al. [83] described that macrophages do not express MC-related OATPs (1a2, 1b1, 1b3, 1c1), but do express others with unknown affinities to MCs.

Thus, as has been evidenced, there are many open questions in relation to cyanotoxins immunotoxicity. Taking into account the pivotal role of the immune system in the homeostasis of all organisms, further research is required to gain knowledge on this important issue. The application of omics, the use of in vitro methods for mechanistic research exploring the potential pathways involved, and in vivo experiments with realistic exposure scenarios could contribute to a better understanding of the complex cyanotoxin pathology.

## Figures and Tables

**Table 1:** In vitro effects of MCs on the immune system.

Cyanotoxin Congener	Experimental Model	Assays	Exposure Conditions	Effects	Reference
MC-LR	Rat astrocytes and macrophages. C6 glial cells	LPS and cytokine-induced production of NO. Expression of iNOS (protein and mRNA level)	2 nM MC-LR for 24 h	It stimulated the LPS- and cytokine-mediated expression of iNOS and production of NO. However, it inhibited the LPS- and cytokine-mediated expression of iNOS and production of NO in rat resident macrophages and RAW 264.7 cells.	[44]
MC-LR	Supernatants from macrophages isolated from Wistar rats	IL-1β and TNF-α levels	0.1, 0.3 and 1.0 µg/mL MC-LR at 0, 40, 50 and 60 min	MC-LR induced the release of IL-1β and TNF-α by peritoneal macrophages in vitro and the supernatants from these macrophages induced electrogenic secretion in rabbit ileal mucosa.	[45]
MC-LR	Human peripheral polymorphonuclear leukocytes	Adherence assays: spontaneous and fMLP stimulated	10^−11^, 10^−10^, 10^−9^ M MC-LR, 8, 25 min	It significantly enhanced the early spontaneous adherence of PMNs but not the late spontaneous adherence or the early or late PMN-stimulated adherence.	[84]
Cyanobacterial bloom samples (containing MC-LR, MC-RR and MC-YR)	Human lymphocytes	Light microscopy, Flow cytometry and analysis of DNA	Lymphocytes were treated with cyanobacterial extract containing MC-LR eq. at 250, 500, 750 or 1000 nM for 24 or 48 h	Morphological changes (apoptosis) and clastogenic effects were observed.	[47]
MC-LR MC-YR	B6C3F1 mouse splenocytes and thymocytes; C57BL/6 mouse thymoma EL-4 cells.	Mitogen-induced lymphoproliferation (LPS and ConA) in splenocytes. IL-2 mRNA expression by RT-PCR, and IL-2 mRNA stability in splenocytes, thymocytes and EL-4 cells	Splenocytes were exposed to 0.1, 1, 10 or 50 μM of each MC-congener for 30 min. Splenocytes, thymocytes and EL-4 cells were exposed to 1 μM of each cyanotoxin for 30 min	Concentration-dependent inhibition of in vitro polyclonal antibody response and lymphoproliferation to LPS were reported. MC-YR decreased Con-A-induced lymphoproliferation. Each MC suppressed IL-2 mRNA expression in splenocytes and thymocytes induced by PMA, but not in EL-4 mouse thymoma cells. MC down-regulated lymphocyte functions and immunosuppression was mediated in part by decreased IL-2 mRNA stability.	[80]
MC-LR	BALB/c mice peritoneal macrophages	NO production, mRNA abundance of iNOS and diverse cytokines using RT-PCR	Stimulation of macrophages with LPS (100 µg/L) and exposure to MC-LR at 1–1000 nmol/L for 24 h	NO production, mRNA levels of iNOS, IL-1β, TNF-α were concentration-dependently decreased, and mRNA levels of GM-CSF and IFN-γ in an independent manner.	[62]
MC-LR	Chicken and human peripheral blood lymphocytes	Lymphocyte proliferation assay, cytokine production (IL-2 and IL-6), and detection of apoptosis	1, 10 and 25 µg/mL MC-LR for 12, 24, 48 and 72 h	At 25 µg/mL MC-LR decreased T-cell proliferation; all concentrations of the toxin inhibited B-cell proliferation. The frequency of apoptotic and necrotic cells increased in a concentration and time-dependent manner. Increased production of IL-6 and decreased production of IL-2.	[75]
MC-LR	BALB/c mice peritoneal macrophages	Expression of mRNA for iNOS and several cytokines (IL-1β, TNF-α, GM-CSF and IFN-γ) by RT-PCR	Induced macrophages with LPS at 1, 10, 100 and 1000 nmol/L C-LR for 6 h	mRNA expression of iNOS, IL-1β, TNG-α, GM-CSF and IFN-γ decreased in comparison to positive control.	[68]
MC-LR	Mouse lymphocytes	Cytotoxicity. Apoptosis by flow cytometry	7.5 µg/mL MC-LR for 4 and 24 h	Decreased the cell viability after 4 and 24 h. Induced apoptosis in mouse B cells, while the T cells were not affected.	[78]
MC-LR and [Asp3]-MC-LR were isolated from a *Microcystis panniformis* strain	Human neutrophils (PMN)	Effects on PMN migration, production of ROS, and phagocytosis and killing assays	PMN migration assay: 0.01–1000 nM of each MC for 60 min. ROS production: 10, 100 nM, for 5 min. Phagocytosis and killing assays: 1 and 1000 nM, for 10 and 15 min	Both MCs induced the production of ROS and increased phagocytosis of *C. albicans*. MC-LR also increased *C. albicans* killing.	[63]
MC-LR MC-RR	Crucian carp (*Carassius auratus*) lymphocytes	In vitro apoptosis assays, using confocal and transmission electron microscopy (TEM), agarose gel electrophoresis and flow cytometry analysis (FC)	Confocal microscopy: 1 nM, 2 h Agarose gel electrophoresis: 1,5,10 nM 2 h TEM: 1 nM 4 h FC: 1,5,10 nM 2 h and 1 nM for 2, 4, 6 and 8 h	Apoptosis even at a low concentration (1 nM) was detected after exposure to both MC-congeners, in a concentration and time-dependent manner. Agarose gel electrophoresis revealed DNA fragmentation caused by apoptosis.	[79]
MC-LR	Rainbow trout (*Oncorrhynchus mykiss*) lymphocytes	Lymphocyte cell viability and proliferation activity assay	Exposure to 1, 5, 10, 20, 40 mg/mL of cell suspension	Concentration-dependent effects of MC-LR on the lymphocyte viability and lymphocyte proliferation.	[92]
MC-LR	Rainbow trout (*O. mykiss*) phagocytic cells	Cell viability assay, leukocyte phagocytosis assay and metabolic activity assay	Cells exposed to 1, 5, 10, 20 µg/mL MC-LR after 2, 4, and 24 h	MC-LR induced time- and concentration-dependent viability decrease. The phagocytic ability was elevated at 5 µg/mL, and MC-LR has also modulatory influence on respiratory burst activity.	[93]
MC-LR MC-LA MC-YR	Rat neutrophils (male wistar rats)	Cell viability and DNA fragmentation assays. Lucigenin-enhanced chemiluminescence assay (ROS release). Cytokine assays by ELISA: IL-8 (human), CINC-2αβ (rat) and TNF-α (human and rat)	1–1000 nM of MCs congeners, for 24 h	Changes in percentage of cells with fragmented DNA after exposure to MC-LA and MC-LR. Increased CINC-2αβ after exposure to MC-LR, and increased extracellular ROS for the three MCs. No significant changes in TNF-α levels and in intracellular ROS levels.	[55]
	Neutrophils from the blood of healthy volunteers			Increased human neutrophils viability after treatment with MC-LR and MC-LA. Increased IL-8 production and extracellular ROS levels.	
MC-LR	Common carp (*Cyprinus carpio* L.) leucocytes	Cytotoxicity studies, respiratory burst activity (RBA test), lymphoproliferation studies and determination of necrotic/apoptotic cells	MC-LR at concentrations of 0.01, 0.1, 0.5 and 1.0 µg/mL	The RBA of phagocytes was increased at 0.01 µg/mL, but it decreased at higher concentrations. MC-LR did not have influence on the T-cell proliferation but decreased the proliferation of B lymphocytes. The toxin induced necrosis to a higher degree than apoptosis.	[94]
Crude MC-containing cyanobacterial extracts; purified MC-containing and no-MC containing extracts	Human lymphocytes	Cytotoxicity by XTT re-duction test, genotoxicity by alkaline comet assay and micronucleus test	Cytotoxicity: incubation for 24h with extracts containing 0-10 µg/mL in culture. Genotoxicity: cells exposed to cyanobacterial extracts with MC between 0-4 µg/mL for 3 and 6h	The highest cytotoxicity and genotoxicity were induced by the purified extract containing MCs (MC-LR, MC-RR and MC-YR). No clear effect with crude extracts. The influence of other compoundsapart from MCs was indated.	[76]
MC-LR	Lymphocytes and phagocytes isolated from the blood of carp (*Cyprinus carpio* L.)	Cytotoxicity, GSH level, DNA fragmentation, caspase-3/7 activities, phagocytosis, effects on the actin and tubulin in the phagocytes	0.01–0.1 µg/mL MC-LR for 2, 6 and 24 h	Pure MC-LR was cytotoxic and induced apoptosis or necrosis in lymphocytes in a time- and concentration-dependent manner. GSH levels did not change in lymphocytes at 6 and 24 h and in phagocytes at 2 h. The toxin induced significant re-organization on the actin and tubulin in phagocytic cells.	[95]
MC-LR	Leucocytes from blood and head kidney from carp (*Cyprinus carpio* L.)	RT-PCR for cytokine gene expression (IL-1β, TNF-α, IL-10, and TGF-β)	0.01–0.1 µg/mL MC-LR for 4 h	MC-LR increased the expression of IL-1β in leukocytes at 0.01 µg/mL, but decreased in head kidney cells at 0.1 µg/mL. The expression of TNF-α mRNA was induced at lower concentrations; in contrast, it was suppressed in blood and head kidney at the highest concentrations. MC-LR, at the highest concentration, increased IL-10 expression, and TGF-β expression was only increased in head kidney cells.	[96]
MC-LR	Human hepatoma cells Huh7	Cytotoxicity, NF-κB activation, levels of TNF-α and IFN-α by ELISA. Gene expression involved in apoptosis, PP2A mRNA by RT-PCR, and protein levels by Western blot. Expression of several INF-α genes by RT-PCR	Cells were exposed to 0.5, 5, 25, 50 µM MC-LR for 6, 24, 48, and 72 h	The expression of NF-κB, IFN-α and several INF-α-stimulated genes was strongly activated. The cytokine TNF-α was also induced. MC-LR induced all endoplasmic reticulum (ER) stress response pathways.	[81]
An extract from a cyanobacterial bloom containing MCs	Lymphocytes and phagocytes from carp blood and head kidney (*Cyprinus carpio* L.).	In vitro: cytotoxicity, apoptosis/necrosis, ROS, lymphocyte proliferation In vivo: Table 2	0.01, 0.1, 0.5 and 1 µg/mL for 24 h	Cytotoxicity at 0.5 and 1 µg/mL. 3-fold higher levels of apoptosis in lymphocytes from 0.5 µg/mL and necrosis at 1 µg/mL. In phagocytes, apoptosis from 0.1 and necrosis from 0.5 µg/mL. ROS changes. Lymphocyte proliferation inhibition in both cell types.	[101]
MC-LR	HepG2 cells and primary mouse hepatocytes (PMHs)	Cytotoxicity, protein content and Western blotting analysis, NF-κB and MAPK, IL-6 production, and ultrastructural changes by transmission electron microscopy	Cytotoxicity: 0–1000 nM of MC-LR for 24 h Protein content: 0–100 nM for 24 h for C-dependent assays and to 25 nM for 3–24 h for time-course assays IL-6: 1–50 nM for 24 h or 20 nM for 6–48 h TEM: 0–100 nMfor 24 h	At noncytotoxic concentrations of MC-LR a proinflammatory effect on hepatocytes was demonstrated by inducing the activation of the NF-κB and MAPK pathways and IL-6 expression in a concentration-dependent manner. Cytotoxic concentrations of MC-LR induced a dysfunction of the NF-κB and MAPK signaling pathways, disruption of mitochondria, and even cell death. PMHs were more sensitive to MC-LR induced cytotoxicity than HepG2.	[82]
MC-LR	Carp (*C. auratus*) lymphocytes	Cytotoxicity assay, oxidative stress parameters (ROS, MDA, GSH, SOD, CAT) and apoptosis	Lymphocytes were incubated with MC-LR (1 µg/L) and different concentrations of quercetin (QE) (0–1000 µg/L), 24 h	Cytotoxicity and ROS formation were suppressed by QE in a concentration-dependent manner. It also enhanced the endogenous antioxidant defense system and the Bax/Bcl-2 ratio. The percentage of apoptosis at the highest QE concentration was lower by nearly half compared with the only MC-LR exposed group, and this concentration inhibited the expression of caspase-3 protein.	[97]
MC-LR	RAW 264.7 murine macrophages	NO production, release of TNFα and IL-6, PP activities, Western-Blot analysis of MC-LR, MAPKs, NF-κB and iNOS	1–1000 nM MC-LR at different times (30 min to 24 h), various combinations of treatments including the activation of cells by LPS	MC-LR-dependent activation of NF-κB and ERK1/2, as well as the production of TNFα can be associated with the stimulation of toll-like receptors (TLRs).	[83]
MC-LR	Bovine Sertoli cells	Morphological changes, cell viability, gene expression by RT-PCR of cytokines and TLR4, TNF-α, NF-κB, etc.	Cells were exposed to 0, 20, 40, 60, and 80 µg MC-LR/L	Nuclear morphological changes and downregulation of the blood-testis barrier constituent proteins within 48 h after treatment. The TLR4 and NF-κB were activated and upregulated, and the proinflammatory cytokines were upregulated within 48 h. At 72 h, upregulation of cytokines and expression of blood-testis barrier constituents proteins were found.	[85]
MC-LR	Bovine Sertoli cells	Mitophagy, mitochondria membrane assessment, gene expression of diverse cytokines and DNA replication related genes by RT-PCR and Western blot analysis	Cells were pretreated with TLR4-IN-C34 (C34) factor for 1 h and exposed to 80 µg MC-LR/L for 24 h	TLR4-IN-C34 attenuated the effects of MC-LR: inhibited mitochondria membrane damage, mitophagy and downregulation of blood-testis barrier constituent proteins via TLR4/NF-κB and mitochondria-mediated apoptosis signaling pathway blockage. Downregulation of the mitochondria electro transport chain, energy production and DNA replication related genes, and upregulation of inflammatory cytokines were modulated by TLR4-IN-C34.	[71]
MC-RR MC-YR	Murine macrophage cell line RAW264.7	Cell viability, NO production and release of TNF-α and IL-6	Cells were preactivated for 2 h by LPS and/or (co)exposed with MC-RR, MC-YR (1–1000 nM) for 24 h	At non-cytotoxic concentrations, both MCs congeners did not have significant effects on macrophage production of pro-inflammatory cytokines (TNF-α and IL-6) neither alone nor with co-exposure with LPS. They had no effect on production of NO.	[77]
MC-LR, MC-RR, MC-LA	Engineered HEK293 cells that utilize an NF-κB-inducible SEAP	Immunomodulatory Compound screening method based on engineered HEK293-hTLR NF-κB assay, to identify activators or inhibitors of specific TLRs	100 nM of each MC	Cells expressing TLR2-9 are not activated by the different MCs, and none of the toxins blocked the ability of TLR2-9 to interact with specific TLR agonists and they are not considered as TLR antagonists. Moreover, TLR negative cells were not stimulated by the assayed toxins. None of the assayed toxins directly interacted with human TLRs in either an agonistic or antagonistic manner.	[86]

CAT: catalase; CINC-2αβ: cytokine-induced neutrophil chemoattractant-2αβ; ConA: concanavalin A; ERK 1/2: Extracellular signal-regulated protein kinases 1 and 2; fMLP: formyl-methionyl-leucyl-phenylalanine; GM-CSF: granulocyte macrophage colony-stimulating factor; GSH: glutathione; Huh7: human hepatoma cells; IL-1β: interleukin-1β; IL-2: interleukin-2; IL-6: interleukin-6; IL-8: interleukin-8; IL-10: interleukin-10; IFN-α: interferon- α; IFN-γ: interferon- γ; LPS: lipopolysaccharide; MAPK: mitogen-activated protein kinase; MDA: malondialdehyde; NF-κB: nuclear factor kappa-light-chain-enhancer of activated B cells; NO: nitric oxide; iNOS: induced nitric oxide synthase; PMA: phorbol 12-myristate 13-acetate; PMHs: primary mouse hepatocytes: PMN: polymorphonuclear leukocytes; PP: protein phosphatases; PP2A: Protein phosphatase 2A; QE: quercetin; qRT-PCR: reverse-transcriptional polymerase chain reaction; RBA: respiratory burst activity; ROS: reactive oxygen species; SEAP: secreted embryonic alkaline phosphatase; SOD: Superoxide dismutase; TGF-β1: transforming growth factor-1; TLRs: Toll-like receptors; TNF-α: tumor necrosis factor- α; XTT: yellow tetrazolium salt; γδ^+^ T: Gamma delta T cells.

**Table 2:** In vivo effects of MCs on the immune system of aquatic organisms.

Cyanotoxin Congener	Experimental Model	Assays	Exposure Conditions	Effects	Reference
MC-LR	Grass carp *(Ctenopharyngodon idella*)	Pathological changes by transmission electron microscopy	Intraperitoneal (i.p.) injection of 50 µg/kg MC-LR body weight (b.w.) Spleen and pronephros were dissected at 1, 2, 7, 14 and 21 days post injection.	Apoptosis was detected only in lymphocytes in the spleen, and pathological changes were also observed in lymphocytes from pronephros (i.e., edematous mitochondria). The recovery occurred at 21 days post injection.	[109]
MC-LR	Zebra fish larvae (*Danio rerio*)	Transcription of essential genes for early lymphoid development (Rag1, Rag2. Ikaros, GATA1, LcK, TCRα) and heat shock proteins (HSP90, HSP70, HSP60, HSP27) by RT-PCR	Larval zebrafish were exposed in glass wares containing 200 or 800 µg/L MC-LR after 72 h post fertilization and fish were sampled at 12, 24, 48, 96 and 168 h after exposure.	Transcription of Rag1, Rag2. Ikaros, GATA1, LcK, TCRα were upregulated at the highest MC-LR concentration assayed. Increased transcription of HSPs.	[118]
MC-LR	Grass carp (*Ctenopharyngodon idella*)	Changes of 6 immune -related genes (TNF-α, IL-1β, Type I IFN, PGRP-L, IgM and MHC-1) by RT-PCR in spleen and head kidney.	i.p. injection of 50 µg/kg MC-LR b.w. and fish were examined at 1, 2, 4, 7, 14 and 21 days post-injection.	The transcription levels of TNF-α, IL-1β, type I IFN, and PGRP-L in both organs were low at all time points, and those of IL-1β were low in the head kidney at different points. IgM and MHC-1 transcription levels were low in both organs studied only at 21 days post injection. MC-LR inhibited immune function at the transcription level.	[110]
MC-LR isolated from cyanobacterial blooms (mainly *M. aeruginosa*)	Bighead carp (*Aristichthys nobilis*)	Expression profile of IL-8 in carps by RT-PCR	Carps were injected i.p. with 50, 200 and 500 µg MC-LR/kg b.w. and sampling times points were 3 and 24 h after injection.	The expression of IL-8 gene in different tissues (liver, kidney, intestine, etc.) was upregulated by MC-LR exposure in a temporal and dose-dependent manner.	[111]
Lyophilized cyanobacteria containing MCs	Carp (*Carassius auratus*)	Morphological and immunological parameters.	Two diets containing 20% and 40% of cyanobacteria lyophilized powder and a control group. 30 days of exposure. The MCs content were: 1.41 mg MCs/g d.w., among which MC-RR, -LR, and –YR were 0.84, 0.50 and 0.07 mg/g dry weight (d.w.)	Significant increases in head kidney and spleen indexes in the high dose group. Marked hemorrhage and hyperemia in kidney and spleen in high dose group. Edematous mitochondria, nucleus deformation and compaction of chromatin in lymphocytes of head kidney and spleen in both exposed groups. Lysozyme activity increased in low dose group but decreased sharply in high dose group. Significant increase of macrophage bactericidal activity in the low dose group.	[106]
MC-LR standard and an extract from a cyanobacterial bloom containing MCs	Lymphocytes and phagocytes from carp blood and head kidney (*Cyprinus carpio* L.).	In vitro: Table 1. In vivo: ROS production by phagocytes, mitogen-stimulated proliferation of lymphocytes and cytokine gene expression.	Control fish and fish exposed by immersion to pure MC-LR (25 µg/L), or to an extract containing 25 µg/L MCs. Fish were sacrificed after 1, 3, and 5 days of exposure.	In vivo, the extract containing MCs had greater suppressive effects on immune cells in comparison to MC-LR standard. After exposure to the extract an up-regulation of IL-1β, TNF-α and IL-10 expression was reported, and there was no impact of the expression of TGF-β. This cytokine was up-regulated in head kidney cells only after 24 h exposure to the extract. The pure toxin only increased IL-1β levels in leucocytes isolated from exposed fish, although to a lower extend than the extract.	[101]
MC-LR	Zebra fish adult (*Danio rerio*)	Cytokines gene expression by RT-PCR, and histology of spleen, gut and gills.	0, 1, 5 or 20 µg/L MC-LR for 30 days.	The transcriptional levels of IFN-1 and IL-8 in spleen were up-regulated at 20 µg/L, and IL-1β and TNF-α increased at 1 µg/L. The mRNA levels of IFN-1, IL-1β, IL-8, TGF-β and TNF-α were increased in intestine and gills in all MC-LR treated fish. Pathological changes were observed in the three organs studied.	[72]
MC-LR	Common carp (*C. carpio*)	Antioxidant enzyme activities, LPO, serum complement C3, lysozyme, bactericidal activity, and gene expression of inflammatory cytokines (IL-1β, TNF-α and IFN I) and heat shock proteins (HSP70 and HSP90).	Four treatment diets: Group I and II fed with control diet; groups III, IV and V fed with 0.5, 1.0 and 2.0 g/kg L-carnitine, respectively. After 4 weeks of feeding, the II-V groups were injected i.p. with MC-LR at dose of 150 µg/kg, and control group with saline solution. Sampling points were set at 0, 12, 24, 48 and 96 h.	MC-LR alone led to a significant downregulation in immune response, including serum complement (C3), lysozyme and bactericidal activity, and increased oxidative stress response. L-carnitine pretreated groups caused elevation in immune response and gene expression of inflammatory cytokines, including heat shock proteins. Antioxidant activities and LPO returned to background levels after L-carnitine pretreatment.	[98]
Cyanobacterial Bloom	Shrimps (*Litopenaeus vannamei*)	Expression profiles of immune genes (RT-PCR), phagocytic activity of hemocytes	Shrimp were sampled at 15, 30, 50 and 70 days post the cyanobacterial bloom occurrence in a shrimp farm (China)	Many antimicrobial peptides genes were down-regulated, whereas the expression of C-type lectins was up-regulated. The concentration of hemocytes in hemolymph was decreased, but their phagocytic activity was increased.	[112]
MC-LR	Male zebrafish (*D. rerio*)	Immunological gene expression, serum immune splenic inflammatory changes and pathology	0–30 µg/L MC-LR for 30 days	At 0.3–3 µg/L MC-LR splenic inflammatory changes were described, including increased serum C3 levels and upregulated expression of innate immune related genes. At 10–30 µg/L MC-LR, degeneration of splenic lymphocytes and macrophages, down-regulation of immune-related genes, and decreased level of serum C3.	[59]
MC-LR	Grass carp	Transcriptome analysis: 457 differentially expressed genes (DEGs) were identified using RNA-Seq. Histopathological study	i.p. injection of 25, 75 and 100 µg MC-LR/kg b.w., and control group. Fish were sacrificed 96 h after injection.	61, 203 and 129 genes immune-related genes were regulated at 25, 75 and 100 µg/kg MC-LR, respectively, indicating a disruption of the immune system. Liver damage induced by MC-LR was also observed.	[107]
Cyanobacterial cells containing MC-LR, MC-RR and MC-YR	Blunt snout bream (*Megalobrama amblycephala*)	Accumulation of MCs in the organs, macrophage phagocytosis and respiratory burst activities, pathological changes in lymphocytes	Fish exposed to NH3-N (0, 0.06, 0.12 mg/L) and fed with diets containing 15 and 30% of toxic cyanobacteria lyophilized powder for 30 days.	NH3-N could promote the accumulation of MC-LR and MC-RR in immune organs of fish (head kidney and spleen). Morphological changes were also detected in head kidney lymphocytes. Significant interaction between cyanobacteria and ammonia exposure on head kidney macrophage phagocytosis activity, respiratory burst activity, number of white blood cells and the transcriptional levels of some immunoglobulins.	[108]
MC-LR	Silver carp (*Hypophthalmichthys molitrix*)	ALT, AST, white blood cells, C3 and lysozyme activity in serum, IgM level, and TNF-α, IL-1β, IFN-γ contents	i.p. injection of ½ and ^1/5^ of 24 h LD_50_ (261.1 and 104.9 µg/kg, respectively) and sacrificed after 6, 9, 12, 24, 72, 168 h.	Increased ALT and AST activities in serum, and the number of leukocytes, complement C3 level, lysozyme activity (9 h) and contents of cytokines. Increased IgM levels were also observed.	[16]
Microcystis aeruginosa cells	Zebrafish (*D. rerio*)	Histological analysis, levels of inter-leukines (IL-1α, IL-1β, IFN-α, and TNF-α). Gene transcription analyses.	Zebrafish were exposed by immersion for 96 h to different concentrations of *M. aeruginosa* cells (low and high densities)	At high concentrations significant increases of cytokine levels were reported, and transcription of inflammatory genes were restrained. Low concentrations promoted the transcription. In intestines, increased goblet cell proliferation, and intestinal desquamation were detected.	[69]
Cyanobacterial Bloom	Shrimps (*Litopenaeus vannamei*)	Expression profiles of immune genes (RT-PCR), phagocytic activity of hemocytes	Shrimp were sampled at 15, 30, 50 and 70 days post the cyanobacterial bloom occurrence in a shrimp farm (China)	Many antimicrobial peptides genes were down-regulated, whereas the expression of C-type lectins was up-regulated. The concentration of hemocytes in hemolymph was decreased, but their phagocytic activity was increased.	[112]
MC-LR	Male zebrafish (*D. rerio*)	Immunological gene expression, serum immune splenic inflammatory changes and pathology	0–30 µg/L MC-LR for 30 days	At 0.3–3 µg/L MC-LR splenic inflammatory changes were described, including increased serum C3 levels and upregulated expression of innate immune related genes. At 10–30 µg/L MC-LR, degeneration of splenic lymphocytes and macrophages, down-regulation of immune-related genes, and decreased level of serum C3.	[59]
MC-LR	Grass carp	Transcriptome analysis: 457 differentially expressed genes (DEGs) were identified using RNA-Seq. Histopathological study	i.p. injection of 25, 75 and 100 µg MC-LR/kg b.w., and control group. Fish were sacrificed 96 h after injection.	61, 203 and 129 genes immune-related genes were regulated at 25, 75 and 100 µg/kg MC-LR, respectively, indicating a disruption of the immune system. Liver damage induced by MC-LR was also observed.	[107]
Cyanobacterial cells containing MC-LR, MC-RR and MC-YR	Blunt snout bream (*Megalobrama amblycephala*)	Accumulation of MCs in the organs, macrophage phagocytosis and respiratory burst activities, pathological changes in lymphocytes	Fish exposed to NH3-N (0, 0.06, 0.12 mg/L) and fed with diets containing 15 and 30% of toxic cyanobacteria lyophilized powder for 30 days.	NH3-N could promote the accumulation of MC-LR and MC-RR in immune organs of fish (head kidney and spleen). Morphological changes were also detected in head kidney lymphocytes. Significant interaction between cyanobacteria and ammonia exposure on head kidney macrophage phagocytosis activity, respiratory burst activity, number of white blood cells and the transcriptional levels of some immunoglobulins.	[108]
MC-LR	Silver carp (*Hypophthalmichthys molitrix*)	ALT, AST, white blood cells, C3 and lysozyme activity in serum, IgM level, and TNF-α, IL-1β, IFN-γ contents	i.p. injection of ½ and ^1/5^ of 24 h LD_50_ (261.1 and 104.9 µg/kg, respectively) and sacrificed after 6, 9, 12, 24, 72, 168 h.	Increased ALT and AST activities in serum, and the number of leukocytes, complement C3 level, lysozyme activity (9 h) and contents of cytokines. Increased IgM levels were also observed.	[16]
Microcystis aeruginosa cells	Zebrafish (*D. rerio*)	Histological analysis, levels of inter-leukines (IL-1α, IL-1β, IFN-α, and TNF-α). Gene transcription analyses.	Zebrafish were exposed by immersion for 96 h to different concentrations of *M. aeruginosa* cells (low and high densities)	At high concentrations significant increases of cytokine levels were reported, and transcription of inflammatory genes were restrained. Low concentrations promoted the transcription. In intestines, increased goblet cell proliferation, and intestinal desquamation were detected.	[69]
MC-LR	Male zebrafish (*D. rerio*)	Histology, serum immune parameters: IL-1β, TNF-α, complement C3. Transcriptions of genes representing the TLR/MyS88 signaling pathway by RT-PCR and western blot assay to measure MyD88 protein levels. Tunnel staining for apoptosis detection.	0, 0.4, 2 and 10 μg/L MC-LR, for 30 days	MC-LR induced increases of serum TNF-α and IL-1β, and a significant upregulated expression of TLR/Myd88 signaling pathway genes. The immunohistochemical and western blot results validated that MC-LR enhanced the MyD88 signal. Significant decreases of serum C3 at the highest concentration was also reported. Pathological changes in spleen, and increased spleen index.	[74]
MC-LR	Zebrafish (*D. rerio*)	Quantification of MC-LR in gonads, eggs, water samples, histological examination, plasma sex hormone measurement, cytokines contents in F1 larvae. Gene transcription by RT-PCR; western blot to measure MyD88 protein levels	Adult zebra pairs were exposed to 0, 0.4, 2 and 10 μg/L for 60 days and the embryos (F1) were hatched without or with continued MC-LR exposure at the same concentrations until 5 days postfertilization (dpf).	Upregulation of innate immune-related genes and increased proinflammation cytokine contents (IL-1β, IL-6, TNF-α) were observed in F1 offspring with/without continued MC-LR exposure.	[70]
MC-LR	Male oriental river prawns (*Macrobrachium nipponense*)	Hormone level analysis, testicular histology examination, sperm quality and testicular antioxidant ability. Gene transcription by RT-PCR and MC-LR levels determination in water samples, embryos and testis. F1 larvae development assay.	0, 0.5, and 5 μg/L MC-LR, for 1, 2 and 4 weeks. F1 embryos did or did not receive continued MC-LR treatment after hatching. The rates of survival, hatching, and malformation in the larvae of the F1 generation prawn after 4 days of exposure to microcystin-LR were determined.	The F1 offspring showed downregulation of immunity molecules (lysozyme, lectin3) and antioxidant molecules, and increased expression of innate immune-related factors (TLR3, MyD88), despite not being treated with MC-LR.	[117]

ALT: alanine aminotransferase; AST: aspartate aminotransferase; b.w.: body weight; C3; complement C3; DEGs: differentially expressed genes; dpf: days postfertilization; d.w.: dry weight; HSPs: Heat shock proteins IFN I: interferon I; IgM: Immunoglobulin M; i.p.: intraperitoneal; IL-1α: interleukin-1α; IL-1β: interleukin-1β; IL-6: interleukin-6; IL-8: interleukin-8; IL-10: interleukin-10; IFN-1: Interferon 1; IFN-α: interferon- α; IFN-γ: interferon- γ; LPO: lipid peroxidation; MHC-I: major histocompatibility complex class I; MyD88: Myeloid differentiation factor 88; NH_3_−N: Ammoniacal nitrogen; PGRP-L: peptidoglycan recognition protein-L; Rag1: recombination activation gen 1; Rag2: recombination activation gen 2; RT-PCR: reverse-transcription polymerase chain reaction; ROS: reactive oxygen species; TGF-β: transforming growth factor; TLRs: Toll-like receptors; TLR/MyS88: MyD88-dependent toll-like receptor; TNF-α: tumor necrosis factor- α; Type I IFN: type I interferon.

**Table 3:** In vivo effects of MCs on the immune system of mammals.

Cyanotoxin Congener	Experimental Model	Assays	Exposure Conditions	Effects	References
Microcystin extract from a bloom	BALB/c Mice	Uptake capacity of peripheral phagocytes. lymphocyte proliferation. Antibody response.	Intraperitoneal (i.p.) injection for 14 days of three doses:16, 32 and 64 mg lyophilized algae cells/kg body weight (b.w.), containing 4.97, 9.94, and 19.88 μg MCs equiv/kg b.w.	Decreased body weight. Spleen and thymus body ratios were altered. A reduction of phagocytosis was reported using phagocytic index of peritoneal phagocytes. Inhibition of LPS-induced lymphoproliferation and a dose-dependent decrease of the numbers of antibody-forming cells were observed. However, no effects on conA-induced T cell proliferation were detected.	[54]
MC extract from cyanobacterial blooms	BALB/c female mice	Expression of multiple cytokines: proinflammatory (IL-1β, TNF-α, and IL-6) and Th1/Th2-related cytokines (IL-2, IL-4 and IL-10) by RT-PCR	Injection of doses of 23, 38, 77, 115 mg lyophilized algae cells/kg b.w., containing 7, 12, 24 and 36 µg MC/kg b.w., respectively.	mRNA levels of some cytokines decreased after injection of all doses, while IL-6 level was unaffected. The level of IL-10 mRNA was transiently up regulated at the lowest dose.	[67]
Blooms containing MC-LR and MC-RR and MC-YR	Rabbits	White blood cell (WBC) numbers and cytokine production (IL-3, IL-4, IL-6, TNF-α, IFN-γ)	Injection (i.p.) of 12.5, 50 µg/kg b.w. Sera were collected from the hearts of rabbits at different times: 0-3 h post treatment in the group of the highest dose, and between 0–168 h post treatment in the group with the lowest dose.	At the high dose WBC number increased but Th1 (TNF-α, IFN-γ) and Th2 (IL-3, IL-4. IL-6) production decreased. In the low dose group the number of WBC and some cytokines production increased in first 12 h, and dropped after 24 h. Some positive correlations between cytokines production were also found.	[119]
MC-LR	Wistar rats	Toxin accumulation. Lysozyme activity, spleen index, histopathological examination and proteome analysis	i.p. injection of MC-LR (1 or 10 µg/kg/day) for 50 days, and control rats.	Significant accumulation in spleen. Decreased lysozyme activity and decreased spleen indexes at the high dose group. Severe damage in spleen and impaired immune functions. 48 splenetic protein levels were modified, mainly involved in immune response, oxidative stress, energetic metabolism and the cytoskeleton assembly.	[121]
MC-LR	Mice (serum, macrophages, leukocytes)	Phagocytosis and ROS of leukocytes, cytokines (IL-6, IL-10 and TNF-α) and DNA-protein crosslink detection (DPC formation)	i.p. injection of 0–25.000 µg/kg/day for 7 days	The level of IL-6 increased with the dose, while TNF-α decreased, and IL-10 had no relationship with the MC-LR exposure. No significant induction of DNA-protein crosslink was detected. The toxin caused a significant dose-dependent counter-effect of phagocytosis for macrophages, but not on the leukocytes.	[122]
Cyanobacterial bloom biomass containing MCs	Peripheral blood, spleen and thymus from Wistar rats	Hematological parameters. Histopathology of spleen and thymus, and lymphocyte populations in peripheral blood, spleen and thymus.	Rats with a diet containing fish meat with and without MCs and complex toxic biomass, for 28 days. Groups D,E: rats fed with diet with 25% of fish (no-MCs) enriched with 700 or 5000 µg total MCs/kg wet weight (w.w.); F: rats fed with diet with 25% of fish from the locality with heavy cyanobacterial bloom.	No histological changes were observed in spleen or thymus. NK cells and γδ^+^ T lymphocytes were increased in peripheral blood in the group exposed to isolated MCs in the food (group E). Significant changes in the ratio CD4^+^ and CD8^+^ cells (increase of CD4^+^ and a drop in CD8^+^) were found in the group F. The greatest changes in lymphoid organs were observed in groups E and F. There was an increase of spleen subpopulations of γδ^+^ T lymphocytes in group E as well as of IgM^+^ lymphocytes (B) and CD8^+^ T lymphocytes.	[120]
MC-LR	Male specific pathogen free (SPF) BALB/c mice	Immune responses in Leydig (LC), Sertoli (SC) and germ cells (GC) were studied, cytokines and chemo-quines in testicular cells, immunohistochemical analysis, sperm counts, q-PCR and western blotting techniques. Spermatogenesis.	Histopathology, Elisa, and western blotting: Mice were i.p. exposed to 20 μg/kg b.w. daily for 7 days. Spermatogenesis: Mice were given drinking water containing 1, 10, 20, or 30 mg/L MC-LR for 90 consecutive days.	MC-LR induced innate immune responses in SC, GC and LC cells. In SC and GC the effects were via the PP2A-dependent phosphatidylinositol 3-kinase (PI3K(AKT/NF-κB) signaling pathway. In LC cells, MC-LR dependent activation of NF-κB and production of proinflammatory cytokines and chemokines may be mediated by Toll-like receptor 2 (TLR2). PI3K(AKT/NF-κB) were also activated in SC, GC, and LC in vivo, with induction of cytokines and chemokines. After chronic exposure decreased sperm counts and abnormal sperm morphology were also reported.	[73]
MC-LR	Male SPF BALB/c mice	Flow cytometry, q-RT- PCR, western blotting, coimmunoprecipitation, Elisa analyses, immuno-histo-chemical analyses, immunofluorescence staining	Mice were given water containing 1–30 μg MC-LR/L for 180 consecutive days.	No apoptosis in Leydig cells (LC) alone. MC-LR can activate macrophages to produce TNF-α and GAS6 in testes, and secreted TNF-α induced apoptosis of surrounding LC. Activated macrophages could engulf apoptotic LC via the Axl-GAS6-PtdSer axis. Reduced serum testosterone levels may be associated with decrease of LCs.	[60]
MC-LR	Male BALB/c mice	SEM, AFM, μ-CT and FTIR in femur bones. Flow cytometry in a suspension of T and B cells from bone marrow, spleen, lymph node and thymus. Levels of cytokines in blood serum (IL-6, IL-10, IL-17A, IFN-γ, and TNF-α)	10 μg/kg b.w. MC-LR day, i.p. for 15 days, and control group. Animals were sacrificed, and bones, lymphoid organs and serum were collected.	MC-LR induced bone loss, impaired lumbar vertebral, tibial and femoral bone micro-architecture, and decreased the mineral density and heterogeneity of bones in mice. MC-LR modulated the population of CD4^+^T, CD8^+^T and B cells, and the toxin increased levels of osteoclastogenic cytokines (IL-6, IL-17A, TNF-α, and decreased the levels of osteoprotective cytokines (IFN-γ, IL-10).	[61]
MC-LR	C57BL/6J mice	Histopathologic study and mRNA expression levels of inflammation factors in jejunum (IL-1β, IL-8, TNF-α, IL-10, TGF- β1)	Oral exposure to MC-LR from drinking water at several concentrations (1, 30, 60, 90, 120 μg/L) for 6 months.	Microstructure of jejunum was destroyed and expression levels of IL-1β, IL-8, TNF-α, IL-10, TGF- β1 were altered.	[123]

AFM: Atomic force microscopy; Axl-GAS6-PtdSer axis: Axl receptor- growth arrest-specific 6- phosphatidylserine axis, b.w.; body weight; ConA: concanavalin A; DPC: DNA–proteins crosslinking; μ-CT: Micro computed tomography; FTIR: Fourier transform infrared spectrophotometer; GAS6: growth arrest-specific 6; GC: germ cells; IgM: Immunoglobulin M; i.p.: intraperitoneal; IL-1β: interleukin-1β; IL-2: interleukin-2; IL-3: interleukin-3; IL-4: interleukin-4; IL-6: interleukin-6; IL-8: interleukin-8; IL-10: interleukin-10; IL-17A: interleukin 17A; IFN-γ: interferon- γ; LC: Leydig cells; LPS: lipopolysaccharide; NF-κB: nuclear factor kappa-light-chain-enhancer of activated B cells; NK cells: natural killer cells; PI3K/AKT/NF-κB: phosphatidylinositol 3-kinase nuclear factor kappa B; PP2A: protein phosphatases 2A; q-PCR: quantitative polymerase chain reaction; qRT-PCR: reverse-transcription polymerase chain reaction; ROS: reactive oxygen species; SC: Sertoli cells; SEM: Scanning electron microscopy; SPF: specific pathogen free; TGF- β1: transforming growth factor β 1; TLR: Toll-like receptor; TNF-α: tumor necrosis factor- α; WBC: White blood cell; w.w.: wet weight; γδ^+^ T lymphocytes: Gamma delta T lymphocytes.

**Table 4:** Effects of CYN on the immune system.

Cyanotoxin	Experimental Model	Assays	Exposure Conditions	Effects	Reference
*C. raciborskii* culture containing CYN	Male mice	Acute oral toxicity assay, including histopathological study	Single oral dose of CYN from a *C. raciborskii* culture containing 0.2% CYN	The median lethal dose was in the range of 4.4-6.9 mg/kg equiv CYN. Death occurred at 2–6 days. The most remarkable changes were observed in the liver, with periacinal coagulative necrosis. Acute renal tubular necrosis, atrophy of the thymic cortex and the lymphoid follicles in the spleen, myocardial hemorrhages, and ulcerations in esophagus and gastric mucosa were also observed.	[50]
CYN	Human peripheral blood lymphocytes	Proliferation lymphocytes	0.01, 0.1,1 µg/mL CYN for 24 h	Significant inhibition of lymphocytes proliferation at the highest concentration assayed.	[53]
CYN	Human peripheral blood lymphocytes	Proliferation assay, cell viability and cell cycle assays	0.01, 0.1, 1 µg/mL CYN after 0, 6, 24, 48 h of the 72 h culture	Cell proliferation was inhibited at 1 µg/mL. CYN affected the viability of human T-lymphocytes in a concentration and exposure time dependent manner. Exposure to 1 µg/mL CYN at the beginning of activation and after 6 h decreased the number of cells entering G2/M phase, and increased number of cells blocked in G0/G1 or prolonged S phase.	[56]
CYN purified from *C. raciborskii*	Human peripheral blood neutrophils	ROS production, phagocytic activity, cell number and viability	0.01, 0.1, 1 µg/mL CYN during 1 h exposure	CYN decreased the level of ROS production in stimulated neutrophils, and in unstimulated cells. CYN did not affect the percentage of phagocytic cells or the number of engulfed bacteria. The toxin did not induce apoptosis or necrosis.	[57]
CYN purified from *C. raciborskii* and non-CYN producing *C. raciborskii*	Human neutrophils and lymphocytes	Viability assays of lymphocytes and neutrophils, and lymphocyte proliferation	Cells were exposed to 0.01, 0.1 and 1 µg/mL of purified CYN. Cells were exposed to *C. raciborskii* cell-free extract	Short term extract treatments altered viability of cells, but CYN did not induce similar effects. Lymphocytes in general were more resistant to cell-free extracts than neutrophils. Differences between lymphocyte responses in isolated and whole-blood cultures were observed. Significant antiproliferative properties were found for the lowest CYN concentration in whole-blood culture. Both extracts exhibited immunomodulatory potencies, so unknown metabolites with a toxic pattern different to that of CYN could be involved.	[58]
Purified CYN	Human lymphocytes	ROS production, cell counts, SOD, GPx, CAT activities, LPO	0.01, 0.1 and 1 µg/mL of purified CYN. ROS evaluated at 0.5, 1, 1.5, 3, 6, 24 and 48 h. Cell counts and oxidative stress biomarkers after 3 and 6 h	Concentration-dependent increase in H_2_O_2_ from 0.5 h with the highest values after 3 and 6 h. At both times SOD and CAT activities decreased and GPx and LPO increased.	[127]
CYN	Leucocyte cell line *Cyprinus carpio* (CLC)	Cell viability, proliferation, apoptosis/necrosis, cell morphology and phagocytic activity	0.1, 1, 10 µg/mL CYN for up to 48 h	1, 10 µg/mL CYN were cytotoxic and altered all parameters. CYN impaired the function of phagocytic cells, their ability to engulf bacteria at the lowest and not cytotoxic concentration.	[128]
CYN	Phagocytic cells from the common carp (*C. carpio*)	Phagocytosis, ROS and nitrogen species production, cytokine expression	0.05, 0.1, 0.5, 1 µg/mL CYN for up to 24 h	Cytotoxicity at 1 µg/mL CYN. Decreased phagocytic activity and changes in actin cytoskeletal structures at 0.5–1 µg/mL Increased ROS and nitrogen species at all tested concentrations. Increased expression in the mRNA level of IL-1β and TNF-α. Expression of the TGF-β gene was elevated at the lowest CYN concentration, whereas the toxin did not induce increases at higher concentrations. No effects on IL-10.	[66]
CYN	Murine macrophage RAW 264.7 cells	Cell viability, NO and ROS production, TNF-α and IL-6, western blot analysis of MAPKs, NF-*κ*B, and iNOS	0.001–1 µM CYN alone or together with cyanobacterial lipopolysaccharide (LPS) for 20, 60 min and 24 h	No effects on viability. CYN alone increased the production of TNF-α which correlated with its effects on ROS production, but it had no effect on either expression of iNOS or several pro-inflammatory mediators (NO and IL-6). Moreover, CYN potentiated the effect of cyanobacterial LPS by induction TNF-α, IL-6, and ROS production, including mitogen-activated protein kinase p38 and expression of iNOS.	[37]
CYN	Engineered HEK293 cells that utilize an NF- *κ*B-inducible SEAP (secreted embryonic alkaline phosphatase) reporter	Immunomodulatory Compound screening method based on engineered HEK293-hTLR NF-*κ*B reporter assay, to identify activators or inhibitors of specific TLRs	100 nM CYN	Cells expressing TLR2-9 were not activated by CYN, and the toxin did not block the ability of TLR2-9 to interact with specific TLR agonists, and thus, the toxin is not considered as TLR antagonist. TLR negative cells (HEK293 parental cells) were not stimulated by CYN.	[86]

CAT: catalase; CLC: carp leucocyte cell line; GPx: glutathione peroxidase; H_2_O_2_: Hydrogen peroxide; Hek293 cells: Human Embryonic Kidney cells; IL-1β: interleukin-1β; IL-6: interleukin-6; IL-10. Interleukin-10; iNOS: inducible nitric oxide synthase; LPO: lipid peroxidation; LPS: lipopolysaccharide; MAPKs: mitogen-activated protein kinases; NF-*κ*B: nuclear factor-*κ*B; NO: nitric oxide; ROS: reactive oxygen species; SEAP: secreted embryonic alkaline phosphatase; SOD: superoxide dismutase; TGF-β: transforming growth factor- β; TLRs: Toll-like receptors; TNF-α: tumor necrosis factor-α.

## Data Availability

No new data achieved from this review.
